# Dengue hemorrhagic fever as a rare cause of chronic immune thrombocytopenic purpura—a pediatric case report

**DOI:** 10.1186/s41182-020-00248-1

**Published:** 2020-07-20

**Authors:** V. Thadchanamoorthy, Kavinda Dayasiri

**Affiliations:** 1 Faculty of Health Care Sciences, Eastern University, Chenkalady, Sri Lanka; 2 Base Hospital Mahaoya, Mahaoya, Sri Lanka

**Keywords:** Dengue, Immune thrombocytopenic purpura (ITP), Steroids

## Abstract

**Background:**

Dengue is a common mosquito-borne infection in tropical countries. Dengue incidence in Sri Lanka is generally showing a rising trend. Both chronic immune thrombocytopenia purpura (ITP) children and chronic ITP triggered by dengue fever in the pediatric age group are rarely reported. This unusual presentation is a diagnostic challenge to clinicians. The authors have reported a pediatric patient who presented with chronic ITP following recovery from dengue hemorrhagic fever.

**Case presentation:**

A 14-year-old previously healthy boy was initially managed as for dengue hemorrhagic fever. Following initial detection of persistent thrombocytopenia at 2 weeks post-discharge, his parents defaulted follow-up for 1 year as he remained asymptomatic. However, 1 year after initial admission, the child re-presented with ecchymotic patches and a platelet count of 30 × 10^3^/cumm. Review of serial blood counts performed during previous hospital admission and by his parents themselves revealed persistent thrombocytopenia over preceding 12 months. Subsequently, the child had an in-depth evaluation. The diagnosis of ITP was confirmed by ruling out differential diagnosis and he was managed as for chronic ITP. His platelet counts showed good response to oral corticosteroids and he is currently being followed up at the pediatric hematology clinic.

**Conclusion:**

While reporting, a 14-year-old boy who developed chronic ITP following dengue hemorrhagic fever, this report highlights importance of frequent monitoring of blood counts to accurately detect and manage critical phase of dengue fever. The report also highlights the value of monitoring platelet counts in post-recovery phase to ensure they have normalized.

## Introduction

Immune thrombocytopenic purpura (ITP) is a rare autoimmune disorder which can be either primary or secondary due to a number of medical disorders [1]. Secondary ITP is known to occur in association with systemic lupus erythematosus [2], anti-phospholipid antibody syndrome [3], immunodeficiency disorders [4], lymphoproliferative disorders [5], viral infections [6], and medications such as quinine and heparin [7]. Immune thrombocytopenia can be either acute or chronic. Acute ITP is more prevalent in children following viral infections and 70-80% of these children recover without treatment [8]. However, a minority of children have persistently low platelets that lead to chronic ITP [9].

Dengue has a wide spectrum of clinical manifestations which range from mild to severe. Dengue fever has been rarely reported as a cause of acute ITP [10]. Similarly, dengue fever has rarely been reported to cause persistent thrombocytopenia [11]. In this report, the authors have described a pediatric patient who following recovery of dengue hemorrhagic fever, developed persistent thrombocytopenia leading to chronic ITP and subsequently, responded well to corticosteroids. The perpetuation of the low platelet count probably occurred through immunological mechanisms, thus characterizing a condition of post-dengue ITP [12].This report highlights the importance of following-up platelet counts until normalization in children who have recovered from dengue.

## Case presentation

A 14-year-old previously healthy boy presented with fever, headache, generalized body aches, and retro-orbital pain for 5 days duration. As he had symptoms of dengue, he underwent dengue NS1 antigen testing and blood counts on day 3 of fever. Complete blood count (CBC) revealed a platelet count of 170 × 10^3^/cumm and white cell count of 5.7 × 10^3^/cumm. Following NS1 antigen was detected positive, he had serial blood counts and supportive care during initial stage as guided by the general practitioner (GP). Patient was advised to have oral fluids approximately 75-100 ml per hour. On day 5 of fever, platelet count dropped to 98 × 10^3^/cumm and white cell count dropped to 4.2 × 10^3^/cumm, and he was admitted for in-patient observation and management. On admission to hospital, he had stable vital signs which included pulse rate of 140 beats per minute, blood pressure—100/70mmg, capillary refill time of less than 2 s, and hematocrit rise of 16.7% from baseline (42% on admission with baseline hematocrit being 36%). During initial 24 h following admission, he developed vomiting and abdominal pain but had no bleeding manifestations.

Physical examination revealed generalized flushing, right hypochondrial tenderness, 3 cm hepatomegaly, and no signs of leakage. Point of care ultrasound revealed a mild pleural effusion and thickened gall bladder wall. Investigations revealed leucopenia (2.2 × 103/cumm), thrombocytopenia (platelet count—68 × 10^3^/cumm), and deranged liver functions (Alanine aminotransferase-88 U/L, Aspartate transaminase-124 U/L). C-reactive protein and renal functions were normal. Subsequently, he was managed as for dengue hemorrhagic fever in high dependency care unit. Platelets further dropped to the lowest count of 6 × 10^3^/cumm on day 7. However, there were no bleeding manifestations during the course of illness. Dengue serology including both IgM and IgG were positive on day 7 of illness. He recovered on day 11 of illness with evidence of rise of platelet counts. The cell indices at discharge on day 11 were white blood cells—7.5 × 10^3^/cumm and platelet counts—52 × 10^3^/cumm. Follow-up was arranged with local GP to review platelet counts in 3 days and patient was also requested for a follow-up appointment at the tertiary care hospital in 2 weeks.

Platelet counts were followed up to ensure complete recovery although platelet count remained at 47 × 10^3^/cumm at 2-week post-discharge review at the tertiary care hospital. He was subsequently admitted as in-patient for further evaluation although no significant abnormality was found. Platelet count at one-month post-discharge was 100 × 10^3^/cumm. Unfortunately, his parents defaulted follow-up as investigations were normal apart from thrombocytopenia and the patient re-presented with multiple purpura for 1 week duration at 1-year post-discharge. The parents had managed by their own to repeat blood counts over period they defaulted clinic follow-up, and the records revealed persistent thrombocytopenia (ranged from 30 to 45 × 10^3^/cumm).

He was investigated in-depth at this point for persistent thrombocytopenia. Average absolute lymphocyte over the period of 1 year was 3.26 × 10^3^/cumm (based on five previous records during the period they defaulted treatment), and the latest lymphocyte count was 2.6 × 10^3^/cumm. Erythrocyte sedimentation rate was 15 mm/1st hour. Anti-nuclear antibody, anti-double standard DNA, and anti-phospholipid antibodies were negative. Mycoplasma, influenza cytomegalovirus, hepatitis B, hepatitis C, human immunodeficiency virus, and Epstein-Barr virus serology were also negative. Past medical history was not supportive of immunodeficiency and baseline immunodeficiency screen (serum immunoglobulins and complement levels) was negative. There was no recent history of MMR (mumps-measles-rubella) vaccination or use of platelet lowering medications prior to first detection of thrombocytopenia. Blood film showed normal morphology of cells including platelets and showed only thrombocytopenia. Bone marrow examination showed megakaryocytes but was otherwise completely normal. The clinical evaluation and investigations ruled out differential diagnosis confirming the diagnosis of chronic ITP secondary due to dengue infection. Since the child had persistent thrombocytopenia for over 12 months, he was managed as for chronic ITP. The child was commenced on corticosteroids and currently being followed-up at pediatric hematology clinic.

## Discussion

Immune thrombocytopenic purpura is an autoimmune disorder characterized by low platelet count and skin-mucosal bleeding [13].There are a variety of viruses implicated in the etiopathogenesis of ITP, especially in children, and include human immunodeficiency virus-1, hepatitis C, varicella-zoster, rubella, influenza, and Epstein-Barr virus [14]. The exact role of viruses in the pathogenesis of this disorder remains ambiguous although ITP typically shadows a viral illness in children. Thrombocytopenia associated with viral infection seems to result both from a reduction in the production of platelets from megakaryocytes and from a decrease in the half-life of platelets. The underlying mechanisms of post-viral immune thrombocytopenia include pathogenic platelet auto-antibodies [15], impaired megakaryocyte function [16], and T-cell mediated platelet destruction [17]. It is believed that viral antigens mimic platelets (molecular mimicry) triggering off formation of platelet auto-antibodies [18].

Pathogenesis of ITP is understood mainly based on adult studies, and evidence regarding pathophysiology of pediatric chronic ITP is scarce. Chronic ITP is diagnosed when thrombocytopenia persists beyond 12 months in patients with acute ITP [19]. Persistently elevated levels of platelet associated IgG are seen with chronic ITP and support the speculation of the mechanisms of persistent immune platelet destruction. Spleen has a crucial role in both antibody production and platelet destruction. Absolute lymphocyte counts at the initial diagnosis of ITP were predictive variables for the development of chronic ITP in children [20]. The risk for chronic ITP was correlated with lower initial lymphocyte counts [18]. In this patient, lymphocyte count remained within low normal range throughout the course. The initial thrombocytopenia in dengue is thought to be due to bone marrow suppression by the virus. Subsequent thrombocytopenia with rash is due to immune-mediated platelet destruction which is supported by demonstration of virus-antibody complexes on the platelet surface in patients with dengue hemorrhagic fever [21]. It has been shown that patients with dengue fever have IgM anti-platelet antibodies [22].

Dengue fever has been reported in patients with ITP [23]. However, the reverse is uncommon and unusual and often leads to diagnostic challenges. It is also theoretically possible that children with co-existing dengue hemorrhagic fever and immune thrombocytopenia are more likely to have bleeding-related complications due to more pronounced thrombocytopenia. Although platelet counts dropped to 6 × 10^3^/cumm in the reported child, no bleeding manifestations were observed and discharged with the platelet of 52 × 10^3^mm/l. The reported child had a rising platelet count (100 × 10^3^/cumm) and was clinically well at 1 month review. Subsequently, the patient defaulted follow-up as he remained well and due to several social reasons even though the parents knew that their child had persistently low platelet counts. Parents repeated platelet counts frequently and presented to hospital in 1 year only after appearance of symptoms. Although this is not an absolutely correct practice in most other countries, most patients in Sri Lanka are able to get complete blood counts done by their own as dengue is endemic and patients often have free access to visit laboratories for basic blood investigations without needing medical consultations. Though this practice obviously reduces their cost of consultation, time they spend in government hospitals and also cross infection from other patients in overcrowding conditions, there is a high probability of delayed presentations to hospital and potentially bring unfavorable outcomes both to the patient and state.

There are only a few case reports of dengue fever which led to ITP and the majority of these reports were from adult patients. In reported cases, thrombocytopenia was treated with intravenous immunoglobulin [24] , and oral or intravenous steroids [11, 25–29]. Boo et al. [11] and Kohli et al. [25] reported pediatric cases that did not respond well to the initial corticosteroid therapy and complicated with intracranial bleeding [11]. Kumar et al. reported an adult patient who showed satisfactory response to intravenous immunoglobulin following initial lack of improvement with intravenous methylprednisolone [24]. However, the cases reported by Bhalla et al. and Leong et al. had an uncomplicated course with good response to corticosteroids.

This case report highlights the importance of early admission to hospital as the reported patient presented during critical phase of dengue hemorrhagic fever despite early diagnosis. This child’s diagnosis of dengue fever was confirmed on day 3 of the illness. The primary care doctor (GP) could have referred him for early in-patient care. However, he may have considered the cost, overcrowding at local hospitals, parents’ wishes, and the fact that dengue patients with low white cell counts are susceptible to nosocomial infections [30]. He decided to give ambulatory care. Many dengue patients worldwide get ambulatory care [31]. However, if the treating doctor takes a calculated risk and go for ambulatory care, the patient has to be monitored and reviewed frequently (current National Guidelines of Sri Lanka for Management of Dengue in Children and Adolescents). For example, CBC has to be reviewed once a day if the platelet count is > 150,000 per cu. mm. Once the platelet count decreases below 150,000 per cu. mm, CBC has to be reviewed twice daily. However, this child’s CBC was repeated on day 5 (not on day 4) and he was admitted while in critical phase of dengue hemorrhagic fever. Frequent review of CBC could have prevented this presentation and potential complications.

The report also strengthened the rationale of following up all patients with dengue fever until the platelet counts normalize. The clinicians should have a broader approach to persistent thrombocytopenia, and malignancies and connective tissue disorders should be ruled out as a priority. Steroids must be commenced only after ruling out hematological malignancies. The key objective in treating ITP is to maintain the platelet count at a level that would not cause major bleeding. It is widely accepted that it is not necessary to treat asymptomatic patients with moderate thrombocytopenia. Intravenous immune globulin (1 g/kg/day for 2 to 3 consecutive days) is used for treating internal bleeding when the platelet count is less than 5000/μl despite corticosteroid therapy for many days, or when there is progressive or extensive purpura. When treatment is required for immune thrombocytopenic purpura, the treatment of choice is oral prednisolone which is given at 1 to 2 mg/kg/day [32].

## Conclusion

While reporting a 14-year-old boy who developed chronic ITP following dengue hemorrhagic fever, this report highlights importance of frequent monitoring of blood counts to accurately detect and manage critical phase of dengue fever. The report also highlights the value of monitoring platelet counts in post-recovery phase to ensure they have normalized.

## Data Availability

The data that support the findings of this case report are available from the Medical Records Department, Batticaloa Teaching Hospital, but restrictions apply to the availability of these data, which were used under license for the current report and so are not publicly available. Data are, however, available from the authors upon reasonable request and with permission of the Medical Records Department, Batticaloa Teaching Hospital, Sri Lanka.

## Abbreviations

CBC: Complete blood count
ITP: Immune thrombocytopenic purpura
